# Phylloplane Biodiversity and Activity in the City at Different Distances from the Traffic Pollution Source

**DOI:** 10.3390/plants11030402

**Published:** 2022-01-31

**Authors:** Kristina V. Ivashchenko, Maria V. Korneykova, Olesya I. Sazonova, Anna A. Vetrova, Anastasia O. Ermakova, Pavel I. Konstantinov, Yulia L. Sotnikova, Anastasia S. Soshina, Maria N. Vasileva, Viacheslav I. Vasenev, Olga Gavrichkova

**Affiliations:** 1 Institute of Physicochemical and Biological Problems in Soil Science, 142290 Pushchino, Russia; 2 Agro-Technology Institute, Peoples Friendship University of Russia, 117198 Moscow, Russia; korneykova-mv@rudn.ru (M.V.K.); 1032197455@rudn.ru (A.O.E.); sotnikova-yul@rudn.ru (Y.L.S.); 1032213405@rudn.ru (M.N.V.); vasenyov@mail.ru (V.I.V.); 3 Kola Science Centre of Russian Academy of Sciences, Institute of the North Industrial Ecology Problems, 184209 Apatity, Russia; anastasiya.soshina97@yandex.ru; 4 Federal Research Center “Pushchino Scientific Center for Biological Research of the Russian Academy of Sciences”, G.K. Skryabin Institute of Biochemistry and Physiology of Microorganisms, 142290 Pushchino, Russia; sazonova_oi@rambler.ru (O.I.S.); phdvetrova@gmail.com (A.A.V.); 5 Faculty of Geography, Lomonosov Moscow State University, 119991 Moscow, Russia; kostadini@mail.ru; 6 Soil Geography and Landscape Group, Wageningen University, 6707 Wageningen, The Netherlands; 7 Research Institute on Terrestrial Ecosystems, National Research Council, 05010 Porano, Italy

**Keywords:** phyllosphere, trees, pollution, potentially toxic metals, microbial functioning, epiphytes, pathogenic microorganisms, basal respiration

## Abstract

The phylloplane is an integrated part of green infrastructure which interacts with plant health. Taxonomic characterization of the phylloplane with the aim to link it to ecosystem functioning under anthropogenic pressure is not sufficient because only active microorganisms drive biochemical processes. Activity of the phylloplane remains largely overlooked. We aimed to study the interactions among the biological characteristics of the phylloplane: taxonomic diversity, functional diversity and activity, and the pollution grade. Leaves of *Betula pendula* were sampled in Moscow at increasing distances from the road. For determination of phylloplane activity and functional diversity, a MicroResp tool was utilized. Taxonomic diversity of the phylloplane was assessed with a combination of microorganism cultivation and molecular techniques. Increase of anthropogenic load resulted in higher microbial respiration and lower DNA amount, which could be viewed as relative inefficiency of phylloplane functioning in comparison to less contaminated areas. Taxonomic diversity declined with road vicinity, similar to the functional diversity pattern. The content of Zn in leaf dust better explained the variation in phylloplane activity and the amount of DNA. Functional diversity was linked to variation in nutrient content. The fraction of pathogenic fungi of the phylloplane was not correlated with any of the studied elements, while it was significantly high at the roadsides. The bacterial classes *Gammaproteobacteria* and *Cytophagia*, as well as the *Dothideomycetes* class of fungi, are exposed to the maximal effect of distance from the highway. This study demonstrated the sensitivity of the phylloplane to road vicinity, which combines the effects of contaminants (mainly Zn according to this study) and potential stressful air microclimatic conditions (e.g., low relative air humidity, high temperature, and UV level). Microbial activity and taxonomic diversity of the phylloplane could be considered as an additional tool for bioindication.

## 1. Introduction

Urban green infrastructures (GIs) contribute considerably to the quality of life in cities by provisioning important ecosystem services, e.g., microclimate cooling, dust deposition, or biodiversity preservation [1,2]. The phyllosphere or the microbial habitat of plant surfaces is an integrated part of GIs, which affects plant health and therefore contributes to the ecosystem services’ supply [3,4]. Leaves are the dominant component of the phyllosphere, and the specific term “phylloplane” applies to the microbiome inhabiting the leaves’ surface. Despite a hostile environment characterized by ultraviolet exposure, extreme relative air humidity and temperature conditions, and their considerable fluctuations, the phylloplane is densely populated by microorganisms.

During recent decades, the phylloplane diversity of bacteria, fungi, and yeasts has been thoroughly investigated by classic cultivation and metagenomics approaches [5,6]. The phylloplane is sensitive to numerous factors the host tree is exposed to and is specific to a host species. In comparison to natural conditions, urbanization coincides with multiple anthropogenic effects and stressors. Urban GIs are subjected to habitat fragmentation [7], mesoclimatic anomalies [8], and maintenance practices [9], and they are exposed to soil and air pollution [10]. Air purification by absorbing dust on leaf surfaces is among the principal ecosystem services of urban GIs [11], whereas the amount and composition of dust deposited on the tree leaf surface is considered an indicator of the pollution level and pollution source [12]. At the same time, the phylloplane of trees exposed to air pollution is negatively affected by direct contact with contaminants. Hence, evidence that leaves’ microbial community differs between urban and nonurban areas is not surprising [13]. However, clear understanding on the factors responsible for microbial property shifts is still pending.

Exposure to environmental pollution is harmful to humans, animals, and vegetation, with health consequences depending on the pollution type, severity of the exposure, and species-specific tolerance [14]. In the worst-case scenarios, long-term pollution can lead to complete degradation of vegetation, resulting in so-called “industrial barrens” [15,16]. A common approach to evaluate the effect of pollutants on biological systems is gradient studies, representing gradual changes in pollution level, e.g., across the sites located at different distances from the pollution source [17,18]. The pollutants’ effect on the phylloplane could be either direct or executed through changes in leaf morphology and physiology [19,20]. For instance, short-term exposure to some air pollutants strongly attenuates stomatal physiology, which in turn increases plant lethality in response to stress [21,22]. Some phylloplane taxa could benefit from the capacity for pollutants’ degradation and their utilization as nutrients [23,24], whereas others could be suppressed due to toxic effects [25]. In London plane trees, substitution of some phylloplane taxa by other more resistant species resulted in no impact of air pollution on alpha diversity [7]. In a manipulation experiment with Ag nanoparticles, the bacterial and fungal leaf community was altered, favoring anaerobic bacteria and stress-tolerant taxa [20]. Pollution-induced alterations of the microbial community may increase the amount of pathogenic genes and the fraction of pathogenic microorganisms in the phylloplane [26,27].

A taxonomic characterization of the phylloplane is not enough to explain the effect of anthropogenic stressors on the phylloplane’s functionality, if microbial activity (i.e., active, potentially active, dormant, and dead units) is not considered. Meanwhile, only active microorganisms drive biochemical processes and determine the microbial community functioning [28]. An active microbiome is that which is currently involved in metabolic processes, e.g., by producing enzymes, responding to the substrate availability, growing, and reproducing. The portion of the active microbiome under stress conditions could be highly reduced when the active microbial biomass is acting inefficiently in terms of energy requirements and substrate utilization (functional diversity) [29,30]. In this regard, the sensitivity of phylloplane activity to environmental quality in cities remains largely overlooked and mainly studied in isolation, from phylloplane taxa in cultures [31,32].

In this study, we investigated how the microbial activity and the taxonomic and functional diversity of the phylloplane of *Betula pendula* responds to air pollution by sampling trees growing at different distances from heavy traffic road in Moscow. *Betula pendula* was selected as it is one of the typical tree species for the region of the investigation, often dominating in the urban GI of cities and characterized by a high potential for particulate matter (PM) accumulation compared to other deciduous species [33]. Road transport is the primary source of air pollution in Moscow [34,35], making a gradient approach particularly suitable for phylloplane sensitivity investigation. We hypothesize that (1) the phylloplane of roadside trees will demonstrate signs of stress (inefficient functioning), (2) the taxonomic diversity of the phylloplane will be different in trees growing at different distances to the road, (3) the fraction of opportunistic microorganisms will increase with an increase of pollution level, and (4) observed changes will be driven by the concentration of particular pollutants associated with traffic.

## 2. Results

### 2.1. Environmental Conditions along the Gradient

**Figure 1:**
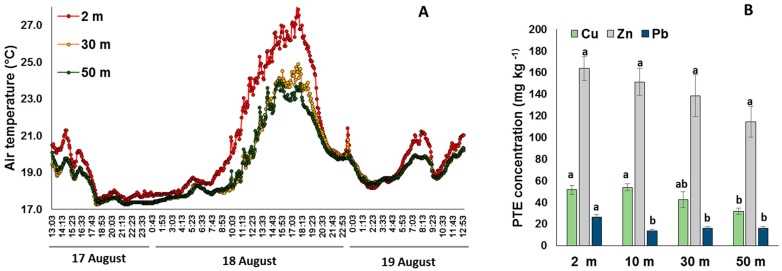
Changes in air temperature hourly from 17 August to 19 August (**A**) and soil pollution (**B**) along the gradient.

A remarkable change of daily average and especially maximal temperature along the gradient was shown even though the sensor from the 10 m distance emergently got corrupted and the data were not available. The daily average temperature at the 2 m distance was 0.8 and 1.0 °C higher compared to 30 and 50 m distance correspondingly, whereas on the afternoon of 18 August 2020 which was the warmest time during the observation period, the difference in air temperature between a distance of 2 and 50 m reached 5 °C (Figure 1A). Significant changes of soil properties along the gradient were also reported, with a gradual decrease in bulk density (from 1.4 ± 0.1 g cm^−3^ to 0.9 ± 0.3 g cm^−3^) and pH (from 7.3 ± 0.1 to 4.7 ± 0.1) with the distance from the road. Over-compaction and neutralization of soil reaction due to dust depositions are indicators of anthropogenic disturbance usually reported for urban soils [36]. Indirectly, an increase in soil organic carbon content (from 3.3 ± 0.3% near the road to 6.0 ± 0.5% in urban forest) confirms higher anthropogenic disturbance in close proximity to the road. A gradual decrease in potentially toxic element (PTE) content on the soil surface with the distance to the road confirms the pollution gradient. Soil bulk content of Cu, Pb, and Zn at 2 m distance from the road was significantly (ANOVA, *p* < 0.05) higher than in the other locations (Figure 1B).

**Figure 2:**
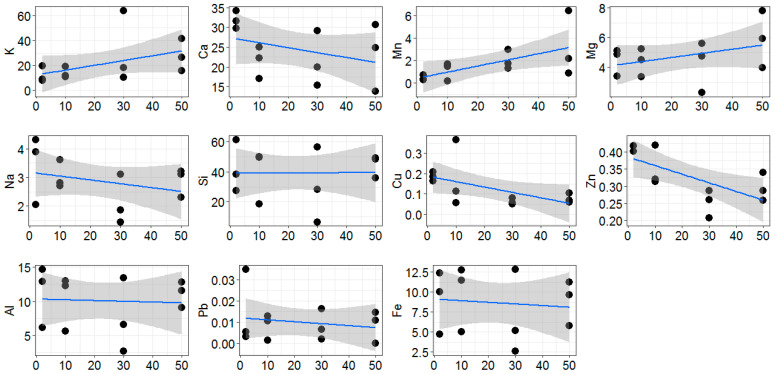
Changing trend of element content in dust collected from the leaf surface (g kg^−1^) in line distance to the road (2–50 m), drawn with smoothing lines (95% confidence interval).

The changing trend in line distance to the road was observed for K, Ca, Mn, Mg, Na, Cu, and Zn (Figure 2). Among them, the content of Cu, Zn, Pb, Fe, Na, and Ca tended to be higher on leaves of the roadside trees (Table 1). Considering the variance of the chemical elements’ concentration on leaves between replicate trees, only Zn was strongly associated with distance to the road, significantly decreasing by 2 times along transects (from the road to the forest sites). In contrast, the concentration of Mn and K increased with distance from the road. For other elements (Al, Si), a clear dynamic in concentration in respect to the distance from the pollution source was not observed.

### 2.2. Phylloplane Activity, DNA Amount, Pathogens, and Diversity

**Figure 3:**
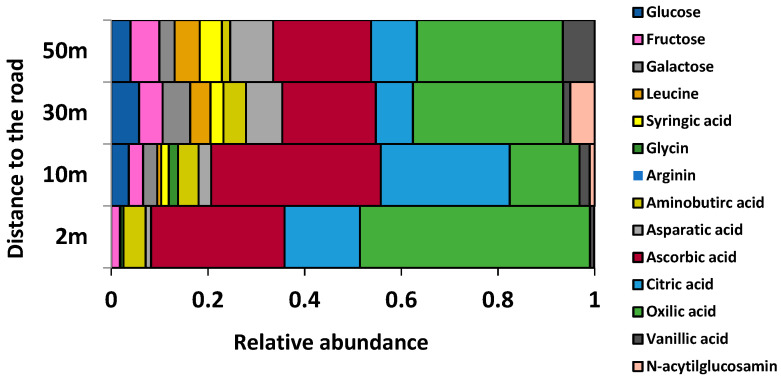
CLPP of the phylloplane at different distances from the road (2–50 m) represented by the contribution of microbial response on various organic substrates.

**Figure 4:**
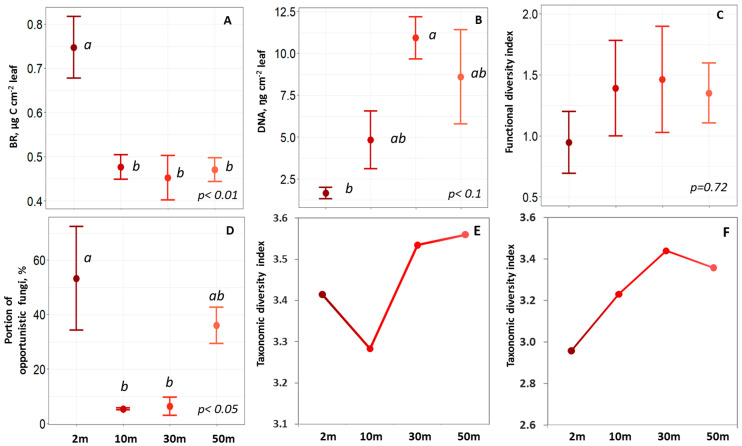
Basal respiration ((**A**), *N* = 3 for each distance site), DNA amount ((**B**), *N* = 3 for each distance site), microbial functional diversity ((**C**), *N* = 3 for each distance site), portion of opportunistic fungi ((**D**), *N* = 3 for each distance site), and Shannon taxonomic diversity index for bacteria ((**E**), *N* = 1 for each distance site) and fungi ((**F**), *N* = 1 for each distance site) of the dust from leaves’ surface at different distances from the road (2–50 m). Data represented by mean (dot) with standard error (whiskers). The mean plot with different letters indicates significant differences between sites (*p* < 0.05).

The phylloplane community-level physiological profile (CLPP) for trees located in roadsides was shifted to domination of the microbial group consuming the most available carboxylic acids: ascorbic, citric, and oxalic (Figure 3). The contribution of these microbial groups gradually decreased with the distance from the roadside. Basal respiration of the phylloplane was 1.5–1.6 times higher at the roadside compared to the sites at the 30–50 m distance (Figure 4A). At the same time, the amount of phylloplane DNA was lower by a factor of 2.9–6.6 for trees at the roadsides in comparison to trees located in the forest, with intermediate rates observed for trees growing at the forest edge (Figure 4B). Microbial functional diversity or the ability to metabolize different substrates tended to decrease along the transect from the road to the core of the forest sites, although variation among the single replicates was considerable, so that no significant differences could be detected (Figure 4C). While the level of total genomic DNA declined closer to the road, the number of cultivable opportunistic fungi and their portion in total fungal diversity substantially increased (Figure 4D, Table 2).

For the roadside trees, 8 species of fungi were identified at 10 m distance from the roadside, 6 species at 30 m and only 3 species at 50 m distance (Table 2). The dominant species in the cultivable fungi community of phylloplane for trees of roadsides were *Ciliciopodium hyalinum* and *P. corylophilum*—at 10 m from the road, only *P. corylophilum*; at 30 m, only *C. hyalinum*—and at the farthest distance *Trichoderma aureoviride* was found. The portion of opportunistic fungi was more than half of the total cultivated fungi community in all sampling points, except for the points at a distance of 30 and 50 m (17–30%). Fungi of the especially dangerous group BSL-2 appeared only at a distance of 2 and 10 m from the road (Table S1). The portion of cultivable pathogenic bacteria in all areas was less than 50% (Table S2).

The diversity index for the microbial community calculated from the sequencing data increased from the roadside to the forest (Figure 4E,F). It indicates the negative effect of traffic on the taxonomic diversity of the microbial community similar to that observed for functional diversity. Such changes in the diversity of fungi were more noticeable (by 11.9%) compared to those for the bacteria community (by 5.6%). This implies that the phylloplane’s fungi diversity is more sensitive to pollution compared to bacteria.

### 2.3. Taxonomic Structure of the Phylloplane’s Microbial Community

**Figure 5:**
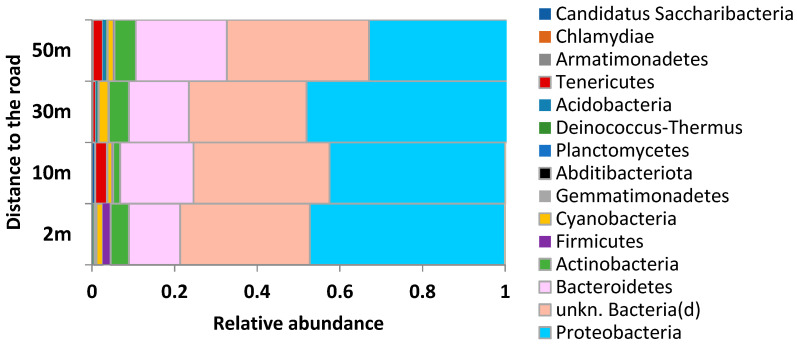
Bacteria community structure (phylum taxonomy level) of the phylloplane at different distances from the road (2–50 m).

*Bacteria.* The analyzed samples contained at least 69,394 reads, and one sample contained 141,475 reads. All high-quality reads were rarefied to get even depths of 61,000 for all samples and binned into operational taxonomic units (OTUs) at 97% sequence identity. A total of 15 identified phyla were detected (Figure 5). Most of the bacteria phyla (8–12) had a relative abundance above 0.1%. The phylum *Proteobacteria* was the most dominant (range 42.5–48.3%), followed by *Bacteroidetes* (12.4–18.5%), *Actinobacteria* (1.7–4.8%), and *Cyanobacteria* (1.1–2.3%). Within the *Proteobacteria*, the most phylotypes were represented by *Alphaproteobacteria*, *Betaproteobacteria,* and *Gammaproteobacteria* (Table S3). *Bacteroidetes* were mostly represented by the classes *Cytophagia, Sphingobacteriia, Flavobacteriia,* and *Chitinophagia.* At the genus level, the most abundant bacterial genera in all studied samples were *Hymenobacter, Sphingomonas, Methylobacterium, f_Oxalobacteraceae,* and *Pseudomonas*, except the dust samples collected 10 m from the road, where *Pedobacter* (*c_Sphingobacteriia*) were also the dominant genus.

**Figure 6:**
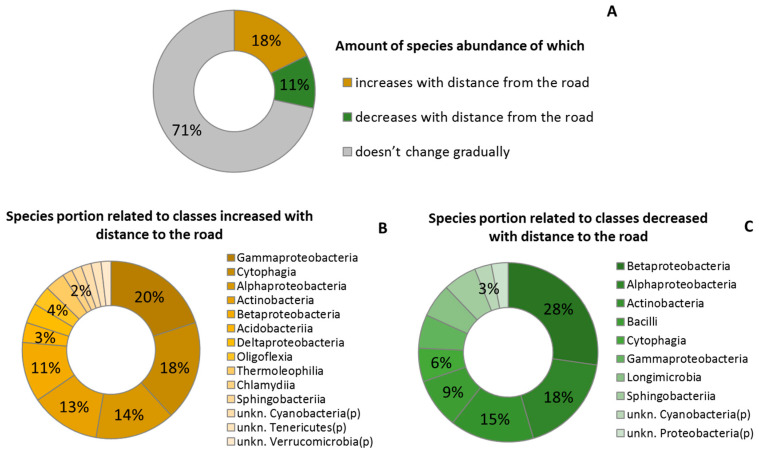
Relative amount of species abundance in relation to road distance (**A**) and breakouts for bacteria classes with increasing (**B**) and decreasing (**C**) abundance.

An extremely high abundance of *Gemmatimonadetes* and *Firmicutes* was found at the closest distance to the road (Figure 5). The abundance distribution of most bacterial species did not relate to road distance (Figure 6A). The number of species that reacted negatively to the traffic (increasing relative abundance from the roadside to the forest) were higher than those that reacted positively. The *Gammaproteobacteria* and *Cytophagia* classes turned out to be sensitive to the pollution, insofar as the abundance of the majority of their species increased from the roadside to the forest (Figure 6B), while *Betaproteobacteria* and *Alphaproteobacteria* classes could be characterized as resistant to the anthropogenic load due to the major portion of their species having the highest abundance on the trees at the roadside (Figure 6C). It should be noted that the majority of species that reacted to the road distance was not dominant in the extracted samples from the phylloplane microbiome (relative abundance < 1%). Among species with high relative abundance (2–8%), distinct increasing and decreasing trends (by 3.6 and 1.5 times) from the roadside to the forest showed unkn. *Pseudomonas* (*g*) and unkn. *Proteobacteria* (*p*), respectively (Table S3).

**Figure 7:**
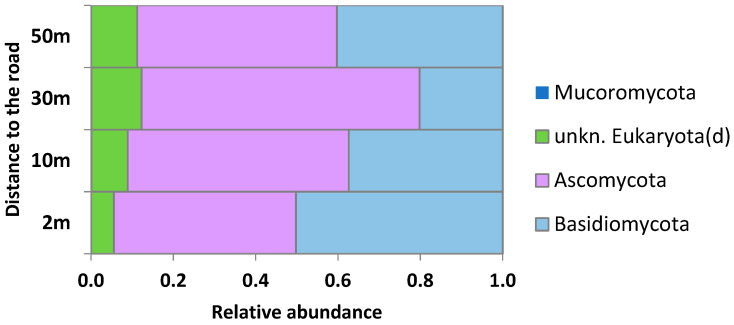
Fungi community structure (phylum taxonomy level) of the phylloplane at different distances from the road (2–50 m).

*Fungi.* The analyzed samples contained at least 39,808 reads, and one sample contained 47,404. All high-quality reads were rarefied to get even depths of 28,000 for all samples and binned into OTUs at 97% sequence identity. The three fungi phyla from four phyla had a relative abundance above 0.1%. *Ascomycota* and *Basidiomycota* in total accounted for more than 88% of all sequencing reads (Figure 7). There were 13 classes of fungi with a relative abundance above 0.1% (Table S4). *Exobasidiomycetes* (*p_ Basidiomycota*) and *Dothideomycetes* (*p_ Ascomycota*) in total accounted for 49% of sequences (44–54%) with a relative abundance above 10%. The most abundant genera (relative abundance 9–23%) were *Microstroma* and *Taphrina* (2 m distance from the road), *Pseudomicrostroma* (10 m), *Ampelomyces* (30 m), and *Pseudomicrostroma* (50 m).

**Figure 8:**
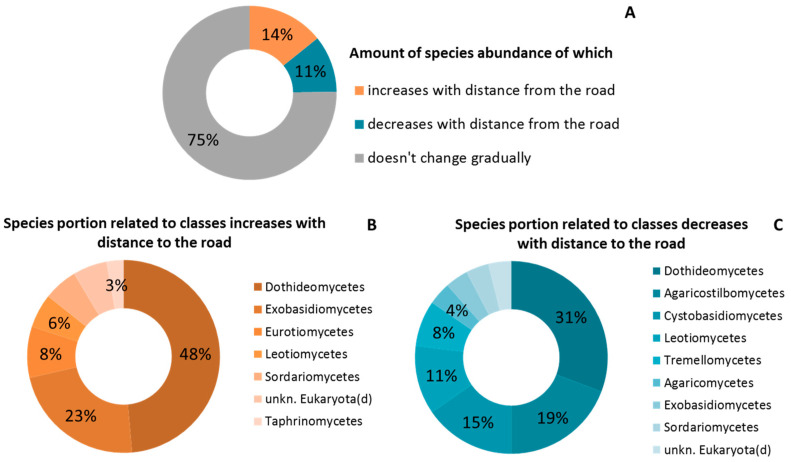
Relative amount of species abundance in relation to road distance (**A**) and breakouts for fungi classes with increasing (**B**) and decreasing (**C**) abundance.

At the phylum level, no distinct distribution trend was found from the roadside to the forest (Figure 8). As was noted for bacteria, the abundance distribution of most fungi species did not change from the roadside to the forest (Figure 8A). At the same time, the portion of fungi species abundance of which was sensitive to the traffic effect was higher than those having the resilience to that. The *Dothideomycetes* class dominated in both groups—those with a decreasing and an increasing trend (Figure 8B,C). The abundance of *Exobasidiomycetes* was almost exclusively characterized by an increase from the roadside to the forest, while the abundance of species belonging to the *Agaricostilbomycetes* class demonstrated a net decline along the gradient (Figure 8B,C). At the species level, the most sensitive to the pollution gradient were *Dothiora sorbi* and *Exobasidium miyabei* (relative abundance increasing from the roadside to the forest by 58 and 1460 times, respectively), and the more resistant were *Kondoa yuccicola* and *Erythrobasidium hasegawianum*, with relative abundance decreasing from the roadside to the forest by 8 and more than 20 times, respectively) (Table S4).

**Figure 9:**
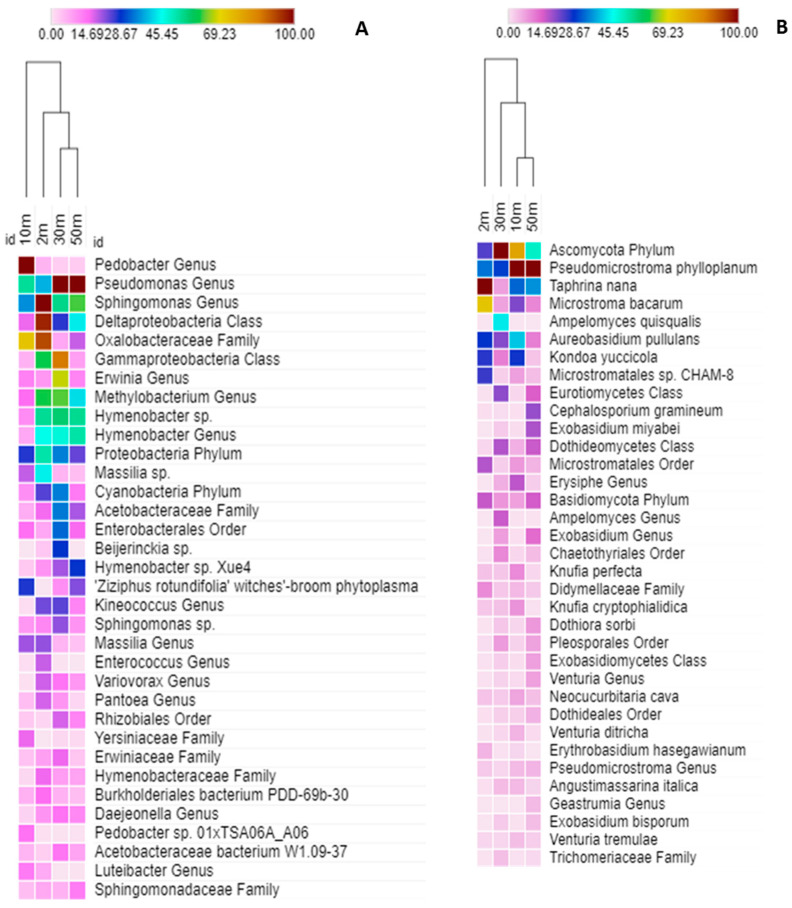
Heat map of the most abundant OTUs of bacteria (**A**) and fungi (**B**) in relation to distance from the road (2, 10, 30, 50 m). Color code from white to brown via cyan (0–45%, 45–100%) depicts relative amount (%) of respective taxonomic group.

The taxonomic structure of the phylloplane’s bacteria community for trees located 10 m from the road differed compared to those at 2, 30, and 50 m distance (Figure 9A). The major similarity was found between bacterial communities for the trees located at 30 and 50 m distance. The taxonomic structure of the phylloplane’s fungi community for trees located 2 m from the road mostly differed from those located at 30, 10, and 50 m (Figure 9B). Hence, the bacteria and fungi community of the phylloplane for trees located 10 m and 2 m from the road differed compared to other studied sites.

### 2.4. Driving Factors of Phylloplane Characteristics

**Figure 10:**
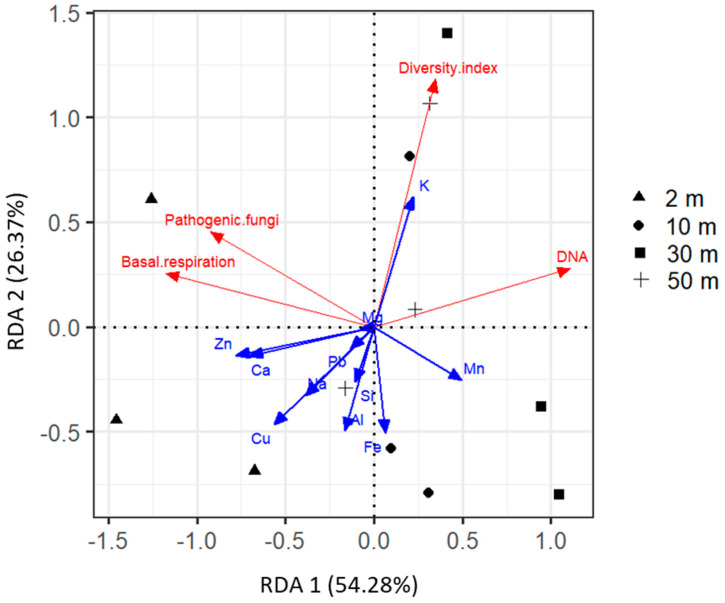
RDA ordination triplot showing the relationships of microbial properties (red arrows) and chemical elements (blue arrows) of the dust from leaves’ surface at different distances from the road (2–50 m).

**Figure 11:**
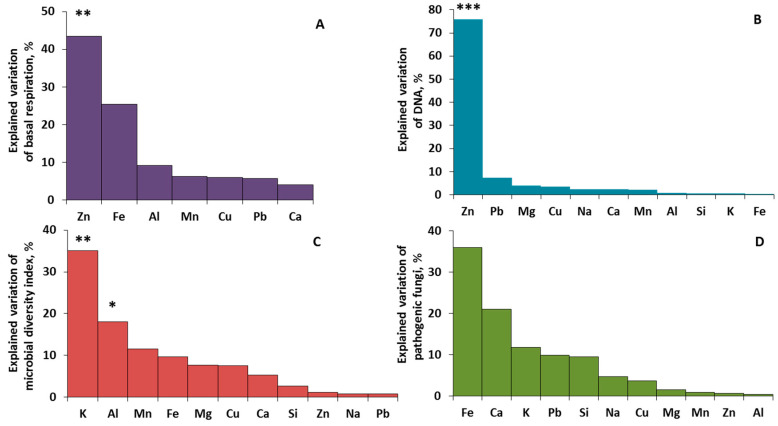
Variance of microbial properties (%), particularly, basal respiration (**A**), DNA (**B**), microbial diversity index (**C**) and pathogenic fungi (**D**), explained by chemical composition (R2 = 0.99 in total) of the dust from leaves’ surface (stepwise linear regression, *N* = 12; ** *p* ≤ 0.05; *** *p* ≤ 0.01; * *p* < 0.1).

Redundancy analysis (RDA) has been used to illustrate variations in phylloplane characteristics (microbial activity, DNA, and pathogen amounts) among the studied sites and the relationships with the concentration of chemical elements. The first two axes together describe 80.6% of total variance (Figure 10). RDA 1 was positively correlated with Mn content (r = 0.50), and negatively correlated with Zn, Ca, Cu, and Na (r = −0.78, −0.71, −0.57, and −0.39, respectively). RDA 2 was positively correlated with the K content (r = 0.62) and negatively associated with Fe and Al (r = −0.51 and −0.50, respectively). The road sites are clearly grouped on the left side of the graph according to RDA 1. According to the angles between the vectors of microbial properties and explanatory variables, it follows that the main predictor for basal respiration and DNA amount is the Zn content (positive and negative effect, respectively), for microbial functional diversity it is K and Al content (positive and negative effect), and for the amount of pathogens no clear driver was found. Detailed stepwise linear regression analysis was used to explain the patterns obtained by RDA. The prevailing portion (43% and 76%) of the explained variance in basal respiration and DNA amount was associated with the Zn content (Figure 11A,B), and 35% and 18% of the variance, respectively, for microbial functional diversity of phylloplane was explained by K and Al content (Figure 11C). For the pathogens the explained variance by studied elements did not rise to the level significance (Figure 11D). The contribution of other studied elements to phylloplane characteristics was poor and nonsignificant.

## 3. Discussion

### 3.1. Environmental Conditions 

Evidence for increases in the concentration of potential toxic elements (e.g., Cd, Cr, Cu, Ni, Pb, Zn) in roadside soils has been reported for Australia [37], Russia [10], China [38], the USA [39], and many European countries [40]. The burning of fossil fuels, consumption of car tires, brake wear, and engine oil are the primary sources of these elements [41]. In the particulate matter collected from the air, Ba, Bi, Cu, Sb, Sn, and Zr have been proposed specifically as traffic-related tracers [42]. Dust collected from the canopy of the roadside trees in 20 countries around Europe was characterized by an increase in the content of Fe and such trace elements as Ti, Cr, Mn, Ni, Cu, Zn, Mo, Sn, and Sb [12]. Instead, the presence in the air and in leaf dust of such elements as Na, Cl, Ca, S Ce, Cs, La, Li, Rb, Sr, and U has been attributed to a soil resuspension process and marine and salt-mine aerosol transfer. Depending on the wind conditions, topography, and physico-chemical characteristic of the substance, the dispersion of the pollutants varies from a few meters from the road to hundreds of meters, with major deposition at a distance of 17–20 m [43,44]. In our study, among the analyzed elements on leaves, a clear negative trend with distance from the pollution source was found for Zn, Cu, Ca, and Na. Zinc enters the roadside dust with tire wear; it is added to the rubber to speed up the vulcanization process. It is used as an anti-oxidizing additive in engine oil, to prevent corrosion in galvanized car body parts, and it is also present in fuel and released with brake wear. Copper is a tracer of brake and tire wear [45]. While Zn and Cu are indicated among typical traffic contaminants, the presence of Ca and Na on roadside leaves is likely due to anthropogenic activity as well, namely due to the dispersion of road de-icing salts in winter [46]. An increase of soil salinity in Moscow roadside soils has been previously reported [46,47]. Resuspension of this soil is subjected to secondary salinization, and its further deposition on leaf blades increases the concentration of Ca and Na in leaf dust. In recent decades, Mn-containing compounds have been added to vehicle technologies, leading to a further increase of Mn concentrations in roadside soils [37]. In our study, Mn concentrations in leaf dust were instead constantly growing with increasing distance from the road. We hypothesize that the soil near the roadside has a lower Mn content compared to the forest soil, so that with a decline in the paved surface a concentration of this element increases in leaf dust due to resuspension. Although not all selected elements exhibit a clear variation along the established transects, the obtained gradient mirrors well the distribution of pollutants between roadside and adjacent green territories.

Alongside pollution, tree isolation and the abundance of paved surface changes the microclimatic conditions to which the phylloplane is exposed in the roadside in comparison to the forest. In this study, the increase of air temperature, especially on clear sunny days, was found to be considerable for roadside trees. The “cool island” effect of green areas surrounding cities is a well-documented phenomenon, showing a strong correlation between forest cover and air temperature [48]. While in this study relative humidity was not measured, its variation is generally negatively coupled to air temperature, hence a gradient in relative humidity could also be expected [49]. Isolated trees are also exposed to higher UV rates—another factor that can impact the microbial community functioning. The UV protection factor is estimated to vary between 4 and 20 for isolated trees, reaching 100 under closed canopies [50].

### 3.2. Phylloplane: Sensitive Indicators to Distance from the Road 

In this study, the activity of the phylloplane turned out to be sensitive to traffic-related air pollution. While the activity of the microbial community of the phylloplane has been largely overlooked, a variety of methods have been proposed to investigate the active portion of the microbiome in soil and water samples [51,52]. The Microresp method was developed for soils, to study the functional diversity of soil microorganisms and its response to variations in environmental conditions [53]. This method was subsequently adopted to study the functioning of aquatic microorganisms [52,54] and to measure pollution-induced community tolerance in waters and soils [29,52]. To our knowledge, the catabolic activity of the phylloplane and its basal respiration has never been evaluated with Microresp, in contrast to the examination of the surface microbial activity in droplet cultures on polystyrene [55,56]. Because the phylloplane shares a portion of the proper microbiome with soils [57,58], it could be expected to show a certain similarity in terms of microbial sensitivity to the pollution load. The specific basal respiration or respiration per unit of biomass of roadside soils was shown to be higher in comparison to control sites in Australia but was not related to the accumulation of any particular metal [59]. In our study, basal respiration was found to be significantly higher for the roadside phylloplane. However, the picture changes once the dimension of the microbial abundance is considered. For the phylloplane, we can take into account its proxy—microbial DNA—similar to some soil-related studies [60], which is five times lower in roadside trees. An increase of the maintenance requirements in the roadside phylloplane could be interpreted as an unstable microbial functioning expressed through high energy consumption per microbial abundance capita in response to pollution and other environmental factors to which the roadside trees are exposed [61]. In other words, higher specific respiration indicates low C use efficiency, meaning that less C is immobilized in microbial biomass, and more C is lost through respiration [62]. Among the analyzed parameters, the Zn content in leaf dust better explains the variation in basal respiration and DNA amount, creating stressful conditions for the phylloplane. A similar effect was demonstrated for soil: high PTE content increased the specific microbial respiration but decreased the microbial abundance [63,64]. Although for microbial cells Zn is an essential micronutrient, required for the stabilization of DNA, RNA, ribosome structure, and enzyme synthesis, its high concentration is toxic for the microbial community. Zn toxicity is executed through the inhibition of proteases, acetate kinases, and coenzyme F420 [65], which lead to a decline in microbial biomass and a depletion of microbial diversity [66,67]. Other studies suggest that high concentrations of Zn lead to a decrease of microbial diversity for both fungi and bacteria [68,69]. In our investigation, Zn content shows a clear trend in line with changes of bacteria and fungi diversity and taxonomic structure of the phylloplane, showing a negative effect on its microbial diversity, corroborating the findings discussed above.

Microbial functional diversity also decreased at the roadsides compared to the forested sites. Its variation was explained by the content of K-nutrient (positive correlation) and Al-PTE (negative correlation). The functional diversity of the roadside phylloplane shifted to the consumers of easily available substrates (carboxylic acids), whereas groups, utilizing more complex aromatic acid compounds (phenolic acids), were less active here. We can suggest the formation of a PTE-tolerant microbial community on the leaves of roadsides, characterized by a low taxonomic diversity and a high metabolic activity in utilizing specific easily available substrates as an energy source [70]. In relation to aromatic compounds—the petrol organic derivatives on leaf surface—many studies demonstrate an increase in their concentration in the roadside environment [10,71,72]. Accordingly, we expected from the roadside phylloplane an enhanced capacity for aromatic ring degradation developed after constant exposure to these pollutants; this, however, was not confirmed.

Among the bacteria, *Pseudomonas* (*g*) turned out to be sensitive to traffic-related air pollution; among the fungi, the species *Dothiora sorbi* and *Exobasidium miyabei* were clearly affected. Several studies have reported negative effects of PTE on fungal growth and reproduction [73,74]. In a study by [75], the presence of Zn did not affect the more represented classes and families of fungi; however, a decrease in Zn negatively affected the amount of less represented OTUs. A summed effect of multiple stressors, anthropogenic and climatological, which interact at tree isolation in the roadside can explain certain species sensitivity to roadside vicinity.

Despite pollutants, roadside trees are characterized by a major exposure to UV due to tree isolation and unfavorable atmospheric conditions (e.g., higher temperatures as measured in this study and low relative air humidity), which can also impact the phylloplane functioning. It has been reported that a microclimatic stress condition influences plant physiology [76,77], which in turn can manipulate the pH level on the leaves’ surface [78]. As is well known, the pH of environmental components (e.g., soil, water) is the driving factor of their microbial activity and taxonomic structure [79,80]. Although we did not measure the pH level for the leaf samples, we cannot exclude the significant influence of this factor on phylloplane structure and activity along the considered gradient.

Summing up, based on our findings, among the sensitive microbial indicators of the phylloplane to air pollution could be named total DNA amount, respiration, and catabolic activity, functional and taxonomic diversity, taxonomic structure, and CLPP. The presence of a certain taxonomic group of fungi or bacteria in the trial is hardly in itself an indicator of air quality because of the uncertain metabolic status and hence contribution to ecological processes of the microorganisms (active vs. dead). Furthermore, the identification could depend on selected primers, bioinformatics, and media in the molecular biology and classic microbiological approaches.

### 3.3. Phylloplane: Resistance to Traffic-Related Air Pollution

During evolutionary changes, microorganisms developed special adaptations to stress conditions, including PTE contamination. The extracellular barrier is an example of the most common and energetically beneficial defense mechanisms and consists of preventing the entry of metal ions into the cell [81]. In addition, there are also intracellular defense mechanisms of microbial cells allowing microorganisms to withstand the PTE presence in the environment. These mechanisms differ depending on the taxonomic group of microorganisms. In bacterial strains, cell resistance to PTE is associated with the ATP activity of their plasma membrane [82]. The melanin pigments of fungi are one of the adaptation mechanisms to environmental stress conditions, enabling the direct binding of the ions of contaminants [83]. The predominance of species of micromycetes containing melanin pigments in soil contaminated with PTE is noted. Their contribution can exceed 50% of the total number of species [84,85,86,87]. In this study, opportunistic bacteria and fungi of the phylloplane were presented in higher abundance at roadside sites compared to forest. For instance, *Enterococcus faecalis* was present only in the roadside phylloplane, evidently being associated with the vicinity of human walking paths. On the other hand, opportunism in microorganisms was demonstrated to be linked to polyextremotolerance [88]. This is because for microorganisms to successfully infect an individual they should be capable of overcoming many protection barriers. Hence, the capability to survive multiple unfavorable factors, such as elevated temperatures, unfavorable pH, humidity, irradiation, and pollutants also provides opportunistic possibilities [89]. An increase in the abundance of some opportunistic bacteria and fungi in roadside trees observed in this study can be explained by resistance of these microorganisms to multiple stressors. Summing up, air pollution obviously impacts the phylloplane taxonomic structure, creating the condition for highly competitive groups which could be presented as opportunistic microorganisms.

Thus, the study demonstrated a considerable effect of traffic on the activity, taxonomic structure, and diversity of phylloplane *Betula pendula*, making it a sensitive indicator of the anthropogenic load. Despite the observed variation in some PTE (Zn, Cu, Na) and the confirmed role of the Zn content in the spatial distribution of microbial respiration activity and DNA content, we cannot exclude the effect of exposures to stressful microclimatic conditions (low relative humidity, high temperature, and UV level) on the phylloplane of isolated roadside trees compared to the “cool islands” of green zones. Future investigations should consider the portion of each climatic factor in phylloplane functioning of different tree species in order to develop recommendations for the improvement of microclimatic conditions from traffic zone landscaping. Particular concern is related to the increase in the fraction of potentially pathogenic species in the phylloplane of roadside trees. Consequently, care must be taken when handling leaves, for example when cleaning areas or working with crowns. It is necessary to ensure the rapid removal of foliage in order to reduce the risks of potential contact to pathogens by sensitive urban populations such as the elderly and children.

## 4. Materials and Methods

### 4.1. Study Site and Sampling

Moscow city is the capital of the Russian Federation and one of the largest urban areas in Europe [90]. The Moscow climate is temperate continental with a mean annual temperature of 5.8 °C and an average annual precipitation of 600 mm. Moscow is located in the southern taiga bioclimatic zone; however, natural vegetation remains mainly in natural protected areas, whereas urban landscapes are dominated by introduced species (i.e., *Tília*, *Pópulus*, *Ácer*, *Castánea*, *Bétula,* etc.). Historically, industrial activities, traffic, and waste disposal have been the main sources of soil contamination by heavy metals in Moscow. In the past several decades, industrial emissions of heavy metals have substantially reduced, but the impact of traffic remains high [34]. Cu, Zn, Pb, and Cd are the prevalent pollutants [91,92]; however, recent studies report a broader range of heavy metals in air and in soils of Moscow [35,93,94].

**Figure 12:**
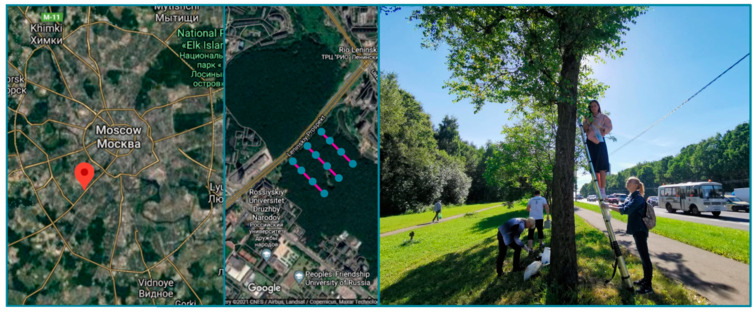
Leaf sampling along the transects.

The negative effect of Leninsky prospect—one of the most heavily trafficked roads in Moscow—on the *Betula pendula* phylloplane was studied. The traffic load was estimated based on the density of the transport flow (number of cars per hour) in morning, noon, and evening periods of the working days of summer. Leaves of *Betula pendula* were sampled along the transects starting from the road and including trees growing 2, 10, 30, and 50 m from the road (Figure 12). Trees growing 2 m from the road were isolated trees without direct contact with other trees, and trees located 10 m from the road belonged to the edge of the urban forest, whereas the trees at the 30 and 50 m distance were inside the forest and were surrounded by other trees. Only healthy trees (class 1 based on a visual tree assessment [95]) belonging to the same age category were selected to reduce heterogeneity.

The tree leaves were collected on 18 August 2020 during the first part of the day (between 10 am and 1 pm), characterized by favorable conditions for plant and microbial functioning in order also to avoid considerable differences in air temperature between studied sites. The last rain event was registered for this part of Moscow 14 days prior to the sampling. The thermal gradient was measured along the 2nd (interim) transect by the autonomous temperature sensor iButton (DS1922) installed at 2 m height with 0.1 ºC accuracy and 5-min steps during the 3-day period (including one day before and one day after sampling).

The gradient in-soil pollution by PTE was measured in situ by the portable X-ray fluorescence analyzer (pXRF) Vanta C. Screening was performed of the three surface samples located within 1 m from the trunk of each of the sampling trees. Soil samples at the locations were collected to measure bulk density, soil organic carbon, and pH (water solution 1:5) as additional indicators of the disturbance level. Leaves were randomly collected from different parts of the canopy at a height between 3 and 4 m above the ground in order to avoid possible disturbance by citizens and to ensure accessibility for sampling [12]. The leaves were placed in sterile bags and delivered to the laboratory, where the preparation of samples for different analytical procedures was started immediately. Leaf area used for each analytical approach was determined by scanning the leaf surface and calculating the area with ImageJ software. 

### 4.2. Chemical Analysis

The leaves’ samples were prepared for chemical analysis in the following way: 30–40 leaves per each site (surface area 96–417 cm^2^_,_ averaged value 265 cm^2^) were placed into a 750 mL flask filled by 50 mL deionized water. The flask with water and leaves was put on a lab rotator and shaken for 15 min at 200 rpm. Then, the dust suspension was poured into a 50 mL flask and kept until complete evaporation of the water at 65 °C for 72 h. Evaporated deionized water was used as a control. The concentration of Al, Ca, Cu, Fe, K, Mg, Mn, Na, Pb, Si, and Zn in the samples was measured using ICP-OES Avio2000 (PerkinElmer, Waltham, MA, USA). The chemical elements listed above were chosen to represent different pollution sources: traffic-origin elements, natural-origin elements, industrial-origin elements [42].

### 4.3. DNA Extraction

In total, 60–70 leaves per each tree (surface area 217–317 cm^2^, average value 269 cm^2^) were mixed with 300 mL of sterile physiological saline solution (8.5 g L^−1^). The obtained suspensions were filtered through Nalgene Rapid-Flow disposable filters with 0.22 PES membrane (Thermo Fisher Scientific, Waltham, MA, USA) to collect the dust deposited on the leaf surface. Then, the membrane filters were cut into small pieces and placed in a Power Bead Pro Tube (QIAGEN, Hilden, Germany). The extraction of DNA from dust deposited on the leaf surface of all samples was performed using the DNeasyPowerSoil Pro Kit (QIAGEN, Hilden, Germany) according to the manufacturer’s protocol. Quantification of DNA was determined using a Qubit 2.0 Fluorometer (Invitrogen/Life Technologies, Carlsbad, CA, USA). DNA was subsequently used as a template for a polymerase chain reaction.

### 4.4. PCR Amplification, Library Preparation, and Sequencing

The PCR amplification, library preparations for next-generation sequencing, and Illumina MiSeq sequencing of the bacterial 16S and fungal ITS rRNA genes were conducted by Sequentia Biotech SL (Barcelona, Spain). The V3–V4 regions of the bacterial 16S rRNA gene sequences were amplified using universal primer pairs 341F–805R [96] including sample-specific barcodes and Illumina sequencing adaptors (Illumina Inc., San Diego, CA, USA). The amplification of the fungal ITS-region was performed using ITS1 and ITS4 primers [97]. After quantification and purification of the PCR products, the amplicon libraries for bacteria and fungi were constructed separately using the 16S RNA Metagenomic Sequencing Library Preparation protocol. Paired-end (PE, 2 × 300 nt) sequencing was performed on an Illumina MiSeq (MiSeq Reagent kit v2, Illumina Inc., San Diego, CA, USA) sequencer following the manufacturer’s run protocols (Illumina Inc., San Diego, CA, USA).

### 4.5. Bioinformatics

Raw read sequences were quality-trimmed while removing adaptor sequences, using Trimmomatic v0.32360. Sequence quality was performed with the FastQC toolkit (Babraham Bioinformatics, Cambridge, UK). A quality check was performed on the raw sequencing data, removing low-quality bases and adapters while preserving the longest high-quality part of the reads. The minimum length established was 50 bp and the quality score 20, which increases the quality and reliability of the analysis. For the taxonomic profiling and quantification of the samples, the proprietary software GAIA (version 2.02, Sequentia Biotech, Spain) was used. GAIA works as follows: (1) each pair of reads is aligned against one or more reference databases, and the best alignments are extracted; (2) a Lowest Common Ancestor (LCA) algorithm is applied to the best alignments; (3) identity and coverage thresholds are applied to the alignments; (4) taxonomy is summarized and reported. The databases used for this analysis included the 16S and the ITS1 + ITS2 sequences obtained from the NCBI “nr” database. All the sequences from each sample were clustered into OTUs based on their sequence similarity (97% identity). Two different alpha diversity metrics (Chao1 richness and Shannon diversity) were calculated on rarefied OTU tables for all samples. Beta diversity was estimated by weighted and unweighted UniFrac distances between samples [98].

### 4.6. Microbial Activity

The 20 sampled leaves (surface area 120–247 cm^2^, average value 180 cm^2^) per each tree were placed in 750 mL flasks in 30 mL of sterile water for preparing the dust suspension for microbial activity analysis and microorganism cultivation (see below). The flasks were placed on a rotator for total mixing of dust suspension for 30 min at 200 rpm. Then, the prepared dust suspension was placed in the two 15-mL sterile flasks that represented the bi-replicate of each sample. The samples of the suspension were kept at +4 °C for maximum 3 days prior to the analysis. The microbial activity was assessed by the Microresp technique [53]. Considering that the technique was developed for soil samples, sterile carbonate-free sand was used for enrichment by dust suspension in the ratio 1:10, ensuring optimal moisture conditions for further analysis. Then, enriched sand samples were placed in a sterile 96-deep well (945 μL volume) and either water or solution of four C-substrate groups was added. In particular, carboxylic (ascorbic, citric, oxalic), carbohydrate (D-galactose, D-fructose, D-glucose), amino- (glycine, L-arginine, L-leucine, α-aminobutyric, L-aspartic), and phenolic (vanillic and syringic) acids were added to the characterized CLPP; water was used to characterize basal respiration (BR). The 96-deep well microplate with the enriched sand was tightly closed in the 96-well microplate with a detection gel and incubated for 6 h at 25 °C. Absorbance by the detection gel was analyzed at a 595-nm wavelength (microplate spectrophotometer FilterMax F5, Molecular Devices, San Jose, CA, USA) before and after incubation and expressed as CO_2_ production in μg C g^−1^ h^−1^ (Moscatelli et al., 2018). Microbial functional diversity was assessed through the Shannon-Wiener index: H = −ΣPi × ln Pi, where Pi is the ratio of respiration response to i substrate addition to total respiration response for all studied substrates.

### 4.7. Microorganism Cultivation

Complementary to metabarcoding, a cultivable portion of phylloplane bacteria and fungi was analyzed. It is known that due to the specificity of the primers used in molecular genetic analysis, not all species of bacteria and fungi can be detected by this method [99]. The main purpose of the plating method was to detect potentially active opportunistic species of bacteria and fungi for which specific nutrient media and a temperature of 37 °C (human body temperature) were used. 

The number of enterobacteria was determined by the plating method on the lactose-peptone medium and Kode’s medium. The number and diversity of cultivable opportunistic fungi was determined using Sabouraud agar nutrient media with the addition of lactic acid (4 mL L^−1^) [100]. The petri dishes were incubated for 2–3 days (for bacteria) and 7–14 days (for fungi) at 37 °C. Microscopic fungi were identified by cultural and morphological characteristics (Olympus CX41 microscope) using standard keys [101,102,103]. To characterize the community structure of cultivable opportunistic fungi, a species abundance index (%), which is equal to the ratio of colonies of a particular species to the total number of colonies, was used. For strains isolated as sterile mycelium, identification was carried out based on the analysis of the region of ribosomal genes ITS1-5.8S-ITS2 rDNA. Sequencing of DNA regions was performed using a BigDye Terminator V. 3.1 Cycle Sequencing Kit (Applied Biosystems, Waltham, MA, USA) with subsequent analysis of the reaction products on an Applied Biosystems 3130l Genetic Analyzer sequencer at the Syntol Research and Production Center (Moscow). The names of fungi were specified according to the updated lists of species in the “Species fungorum” database (www.indexfungorum.org, last accessed 15 November 2021). The portion of opportunistic fungi was calculated from the whole cultivated fungi community that represent the group of pathogens that plague vulnerable individuals with low immunity status [104] (Richardson, 1991). The fungi were classified as opportunistic according to the Hoog classification [105]. Opportunistic fungi were divided into three groups, according to their potential hazard to human health: BSL1, BSL2, and BSL3, representing increasing degree of pathogenicity.

Thus, in this study, the chemical and microbiological analyses were performed by flushing samples from both the upper and lower leaf surfaces.

### 4.8. Statistics

The leaf area and amount of dust for each type of analysis were determined. The calculations for chemical properties were performed per leaf surface (cm^2^) and dust mass (kg), characterizing pollutant quantity and quality, respectively [27]. The same approach was used for microbial properties. Meanplots were used to show the measure of central tendency distribution along the transect from roadside to forest. Descriptive statistics were used to determine the mean and standard error. Significant differences in the variables between the studied sites were examined by one-factor analysis of variance (ANOVA) with Tukey’s multiple comparison test. Prior to the analysis, variance homogeneity was checked by the Levene’s test. RDA was used to (1) show the total variance of microbial properties across all sites; and (2) test the relationships between the studied variables. The predictor variables (concentration of the chemical elements) explaining a variance of microbial properties were assessed by stepwise linear regression analysis with 999 permutations of residuals to test the significance level. Prior to RDA and multiple regression, both dependent and predictor variables were transformed (log base 10-transformation for BR and portion of pathogenic fungi) according to normal distribution adjustment. Additionally, the predictor variables in RDA were the scale to unit variance. Statistical analysis and visualization of experimental data were performed in R.

## Figures and Tables

**Table 1:** Mean concentration of chemical elements (ELM, g kg^−1^) with standard errors for the dust collected from the leaf surface. The values with the different letters significantly differ (*p* < 0.05).

ELM	Distance to the Road, m				*p*-Value
	2	10	30	50	
K	12.1 ± 3.6 a	13.9 ± 2.6 a	30.8 ± 16.8 a	27.8 ± 7.4 a	0.43
Ca	31.9 ± 1.3 a	21.4 ± 2.4 a	21.5 ± 4.0 a	23.1 ± 4.9 a	0.18
Mn	0.5 ± 0.1 a	1.1 ± 0.5 a	2.0 ± 0.5 a	3.2 ± 1.7 a	0.25
Mg	4.4 ± 0.53 a	4.4 ± 0.5 a	4.2 ± 0.9 a	5.9 ± 1.1 a	0.49
Na	3.4 ± 0.7 a	3.0 ± 0.3 a	2.1 ± 0.5 a	2.8 ± 0.3 a	0.35
Si	42.4 ± 10.0 a	39.5 ± 10.4 a	30.4 ± 14.5 a	44.6 ± 4.4 a	0.79
Cu	0.2 ± 0.01 a	0.2 ± 0.09 a	0.06 ± 0.009 a	77 ± 14 a	0.22
Zn	0.4 ± 0.01 a	0.4 ± 0.04 ab	0.3 ± 0.02 b	0.3 ± 0.02 b	<0.01
Al	11.2 ± 2.6 a	10.3 ± 2.4 a	7.5 ± 3.1 a	11.2 ± 1.1 a	0.68
Pb	0.01 ± 0.01 a	0.008 ± 0.003 a	0.008 ± 0.004 a	0.008 ± 0.004 a	0.86
Fe	9.0 ± 2.3 a	9.7 ± 2.4 a	6.8 ± 3.1 a	8.9 ± 1.62 a	0.85

**Table 2:** Cultivated opportunistic fungi species and species abundance index (%) at different distances from the road (2–50 m).

Fungi Species	Distance from the Road, m			
	2	10	30	50
*Aspergillus flavus* Link	5.41	14.58	ND	ND
*A. fumigatus* Fresen.	2.70	4.17	ND	ND
*A. ochraceus* G. Wilh.	2.70	ND	ND	ND
*Ciliciopodium hyalinum* Dasz.	45.95	ND	58.33	ND
*Paecilomyces variotii* Bainier	2.70	ND	ND	ND
*Parasarocladium breve* (Sukapure & Thirum.) Summerb., J.A. Scott, Guarro, & Crous	ND	ND	8.33	ND
*Penicillium citrinum* Thom	ND	ND	8.33	ND
*P. corylophilum* Dierckx	27.03	72.92	ND	ND
*P. glabrum* (Wehmer) Westling	2.70	ND	8.33	ND
*P. restrictum* J.C. Gilman & E.V. Abbott	ND	4.17	ND	ND
*Sterilia mycelia* white	ND	ND	8.33	ND
*Talaromyces helicus* (Raper & Fennell) C.R. Benj.	10.81	ND	-	25.00
*T. ruber* (Stoll) N. Yilmaz, Houbraken, Frisvad, & Samson	ND	ND	8.33	ND
*Trichoderma aureoviride* Rifai	ND	2.08	ND	62.50
*T. viride* Pers.	ND	2.08	ND	12.50

ND, not detected.

## Data Availability

Data set available on request to corresponding authors.

## Supplementary Materials

The following are available online at https://www.mdpi.com/article/10.3390/plants11030402/s1, Table S1: Portion of opportunistic fungi in total amount of fungi (%); Table S2. Cultivable bacteria distribution along transect from roadside to the forest (2–50 m); Table S3: The abundance (operational taxonomic units, OTUs) and relative abundance (%) of bacteria of dust collected from the leaf surface at different distances to the road (2, 10, 30, 50 m); Table S4: The abundance (operational taxonomic units, OTUs) and relative abundance (%) of fungi of dust collected from the leaf surface at different distances to the road (2, 10, 30, 50 m).
