# Supplementary material for: Phylloplane Biodiversity and Activity in the City at Different Distances from the Traffic Pollution Source
Source: Plants (Basel). 2022 Jan 31;11(3):402. doi: 10.3390/plants11030402 (PMC8839900; doi:10.3390/plants11030402)
Supplement: Supplementary file 1 [file plants-11-00402-s001.zip › Table S1.pdf]

Table S1 Portion of opportunistic fungi in total amount of fungi (%)

Distance, m	Cultivated fungi			Sequenced ITS of fungi		
	opportunistic		non-opportunistic	opportunistic		non-opportunistic
	BSL2	BSL1		BSL2	BSL1	
2	37	25	38	0.01	7	93
10	33	33	34	0.01	9	91
30	0	17	83	0.15	5	95
50	0	30	70	0.03	3	97
