# Supplementary material for: Phylloplane Biodiversity and Activity in the City at Different Distances from the Traffic Pollution Source
Source: Plants (Basel). 2022 Jan 31;11(3):402. doi: 10.3390/plants11030402 (PMC8839900; doi:10.3390/plants11030402)
Supplement: Supplementary file 1 [file plants-11-00402-s001.zip › Table S2.pdf]

Table S2. Cultivable bacteria distribution along transect from roadside to the forest (2-50 m)

Distance to the road, m	Total number of bacteria, mln/g	Number of pathogenic bacteria, mln/g	Portion of pathogenic bacteria, %
2	15.3	4.1	27
10	8.4	2.0	24
30	3.0	0.5	17
50	3.6	0.8	22
